# Role of Protein Kinases in Hedgehog Pathway Control and Implications for Cancer Therapy

**DOI:** 10.3390/cancers11040449

**Published:** 2019-03-29

**Authors:** Valentina Montagnani, Barbara Stecca

**Affiliations:** Core Research Laboratory–Institute for Cancer Research, Prevention and Clinical Network (ISPRO), 50139 Florence, Italy; valentina.montagnani@ittumori.it

**Keywords:** Hedgehog, GLI, Smoothened, protein kinases, phosphorylation, cancer, targeted therapy

## Abstract

Hedgehog (HH) signaling is an evolutionarily conserved pathway that is crucial for growth and tissue patterning during embryonic development. It is mostly quiescent in the adult, where it regulates tissue homeostasis and stem cell behavior. Aberrant reactivation of HH signaling has been associated to several types of cancer, including those in the skin, brain, prostate, breast and hematological malignancies. Activation of the canonical HH signaling is triggered by binding of HH ligand to the twelve-transmembrane protein PATCHED. The binding releases the inhibition of the seven-transmembrane protein SMOOTHENED (SMO), leading to its phosphorylation and activation. Hence, SMO activates the transcriptional effectors of the HH signaling, that belong to the GLI family of transcription factors, acting through a not completely elucidated intracellular signaling cascade. Work from the last few years has shown that protein kinases phosphorylate several core components of the HH signaling, including SMO and the three GLI proteins, acting as powerful regulatory mechanisms to fine tune HH signaling activities. In this review, we will focus on the mechanistic influence of protein kinases on HH signaling transduction. We will also discuss the functional consequences of this regulation and the possible implications for cancer therapy.

## 1. Introduction

Hedgehog (HH) signaling is a highly conserved pathway playing an essential role in embryonic development, tissue homeostasis and stem cell maintenance. Its misregulation leads to a number of human disorders, including cancer [1]. Therefore, the activity of the HH pathway has to be tightly controlled at multiple levels. Protein phosphorylation is the most investigated post-translational modification, and it is mediated by protein kinases (PK) and protein phosphatases [2]. PKs transfer a phosphate group from ATP to a substrate protein at serine, threonine or tyrosine residues, whereas phosphatases remove these phosphates. Phosphorylation status of a protein is controlled by a balance between kinase and phosphatase activities, and affects protein conformation, stability, activity and interaction with other proteins [3]. The activity of the HH signaling is regulated by a number of phosphorylation events that occur mainly at the level of the G-protein coupled-receptor (GPCR) SMOOTHENED (SMO) and downstream on the GLI transcription factors, the final mediators of the HH pathway [4]. Here, we will review studies of PKs involved in the regulation of the HH signaling with a focus on cancer. We will also discuss the mechanistic and functional consequences that phosphorylation events play in HH signaling transduction and the implications for cancer therapy.

## 2. Hedgehog Pathway: Principle of Signal Transduction

The Hedgehog pathway has been first identified in *Drosophila melanogaster*, where it is required for proper embryonic patterning and development [5]. Some of the core components of the HH pathway have maintained their function in the evolution from flies to vertebrates, whereas others have substantially diversified. *Drosophila* has only one Hh and one Gli protein, Cubitus interruptus (Ci), whereas in mammals there are three HH family members, Sonic (SHH), Indian (IHH) and Desert (DHH) Hedgehog, and three GLI proteins (GLI1, GLI2 and GLI3) [1]. Distinct from *Drosophila* membrane-mediated Hh pathway, mammalian HH signaling is mainly transduced in the primary cilium (PC) [6], a solitary microtubule-based membrane protrusion that functions as an antenna to sense extracellular cues.

**Figure 1:**
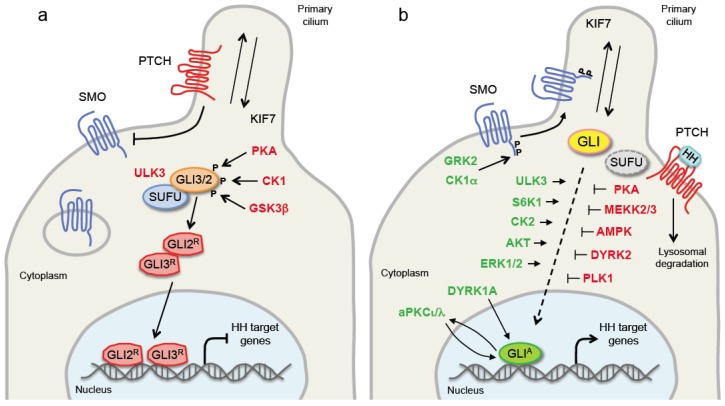
Overview of Hedgehog pathway in absence (**a**) and in presence (**b**) of HH ligand. Schematic diagram of the basic components of the HH signaling (filled circles) and protein kinases that act as positive (green) or negative (red) regulators. GLI2 and GLI3 move within the PC together with KIF7, a member of the kinesin family of anterograde motor proteins. See main text for details. Abbreviations: PTCH1, Patched 1; SMO, Smoothened; SUFU, Suppressor of Fused; HH, Hedgehog; KIF7, kinesin family member 7; CK1, caseine kinase 1; CK2, casein kinase 2; GSK3β, glycogen synthase kinase 3β; PKA, protein kinase A; ULK3, Unc-51 like kinase 3; GRK2, G-protein coupled receptor kinase 2; S6K1, ribosomal protein S6 kinase 1; AKT, protein kinase B; ERK1/2, extracellular signal-regulated kinases 1/2; DYRK, dual specificity tyrosine-phosphorylation-regulated kinase; aPKCι/λ, atypical protein kinase Cι/λ; AMPK, AMP-activated protein kinase; MEKK2/3, mitogen-activated protein kinase kinase kinase 2/3; PLK1, polo-like kinase 1.

PATCHED (PTCH) is a twelve transmembrane protein that functions as the HH receptor and acts as negative regulator of the HH pathway by inhibiting the GPCR seven-transmembrane protein SMO, the signal transducer. In absence of HH ligand, PTCH1 localizes in the primary cilium, where it inhibits accumulation of SMO [7]. Thus, GLI2 and GLI3 are retained in the cytoplasm by the negative regulator of the pathway, Suppressor of Fused (SUFU), and sequentially phosphorylated by protein kinase A (PKA), casein kinase 1 (CK1) and glycogen synthase kinase 3β (GSK3β). These phosphorylation events regulate the proteolysis of GLI2 and GLI3 to C-terminally truncated repressor forms (GLI2^R^ and GLI3^R^) by recruiting the β-transducin repeat containing protein (β-TRCP) E3 ubiquitin ligase [8,9,10]. Binding of HH ligand to PTCH1 initiates the HH signaling. PTCH1 exits the primary cilium, releases the inhibition on SMO, and allows the translocation of SMO into the PC. Thus, active SMO triggers an intracellular signaling cascade promoting the activation of GLI2 and GLI3. Dissociation of GLI2 and GLI3 from SUFU leads to the formation of fully activated GLI2 and GLI3 (GLI2^A^ and GLI3^A^), which translocate into the nucleus where they begin transcription of HH pathway target genes, including GLI1 (Figure 1).

The GLI proteins are members of the Kruppel-like family of transcription factors (TF). All three GLI TF contain five conserved C2H2 zinc-finger DNA binding domains and a histidine/cysteine linker sequence between the zinc fingers. The GLI TF bind the promoter of target genes at the consensus sequence 5′-GACCACCCA-3′ [11], with cytosines in position 4 and 6 essential for the transcriptional activation of target genes [12]. The activation domain is in the C-terminal and is common to all three GLI factors; whereas the repressor domain is in the N-terminal region, and is present only in GLI2 and GLI3 proteins. Therefore, GLI2 and GLI3 act as activators of transcription in their full-length forms, or as repressor forms when truncated by processing. On the other hand, GLI1 acts as an activator of transcription and is induced by GLI2 and GLI3, and further amplifies the initial response of the HH signaling. For this reason GLI1 is considered the best read-out of HH pathway activation. PTCH1 and HH interacting protein 1 (HHIP1) are also targets of the HH signaling, and act as negative regulators limiting the extent of HH signaling at transcriptional level. In addition, the GLI TF control a number of context-dependent targets that are involved in several cellular responses, including proliferation and differentiation, cell survival, self-renewal, angiogenesis, epithelial-mesenchymal transition and invasiveness.

Several mechanisms of aberrant activation of HH pathway have been described in cancer. Ligand-independent activation occurs mainly in basal cell carcinoma (BCC) and in medulloblastoma. It is due to mutations or amplifications of key components of the HH pathway, which induce its constitutive activation, such as loss-of-function mutations in PTCH1 [13] or SUFU [14], two negative regulators of HH signaling, activating mutations in SMO [15], gene amplification of GLI1 and GLI2 [16,17]. This type of aberrant HH pathway activation was found for the first time in patients with Gorlin syndrome, a condition predisposing to cancer due to mutations in the *PTCH1* gene. Ligand-dependent mechanism is characterized by the presence of HH ligands that activate the pathway and it can be autocrine, paracrine and reverse-paracrine. The autocrine pattern, in which tumor cells secrete and respond to HH ligands, has been reported in several cancer types, including lung, pancreas, gastrointestinal tract, prostate and colon cancers, glioma and melanoma [18,19,20,21,22,23,24,25,26,27]. In the paracrine pattern, HH signaling is activated in the stroma by HH ligands secreted from cancer cells. This mode of activation was shown in human xenograft models of pancreatic and colorectal cancers [28]. In the reverse paracrine mode, the tumor microenvironment secretes HH ligands, which activate the HH pathway in tumor cells. Examples of this type of HH pathway activation are represented by experimental models of glioma [29] and hematological malignancies [30,31]. Another mode of aberrant HH pathway activation (non-canonical activation) is engaged by a variety of tumorigenic inputs and signaling pathways, such as the RAS-RAF-MEK-ERK cascade, PI3K-AKT, mTOR/S6K1 or loss of tumor suppressors, which directly or indirectly activate the GLI transcription factors independently of upstream PTCH1/SMO [32,33].

## 3. The Major Phosphorylation Events in Hedgehog Pathway: The Role of Protein Kinases

### 3.1. PKA, CK1 and GSK3β

Protein kinase A (PKA), glycogen synthase kinase 3β (GSK3β) and casein kinase 1 (CK1) are serine/threonine protein kinases, which work in conjunction in the regulation of HH signaling. PKA is a tetrameric complex whose activity depends on cellular levels of cyclic AMP (cAMP) [34]. The CK1 family is involved in several cellular processes, including regulation of Wnt and HH signaling pathways, and consists of seven members (α, β, γ1, γ2, γ3, δ, and ε) [35]. GSK3β acts preferentially on primed substrates [36].

In *Drosophila*, PKA can promote Hh signaling by phosphorylating the C-terminus of Smo at three sites, priming Smo for phosphorylation by CK1. Phosphorylation of Smo promotes its cell surface accumulation and active conformation [37]. In line with this finding, a report in *Drosophila* showed that in presence of Hh, a catalytic subunit of PKA forms a complex with Smo to phosphorylate its C-tail, inducing its conformational change and oligomerization, leading to Hh pathway activation [38]. A recent study confirmed a positive role of PKA in HH pathway also in mammalian cells, revealing the Ptch1-ArhGAP36-PKA-Inversin axis as a mechanism for inducing SMO ciliary translocation and, hence, HH pathway activation [39].

PKA, GSK3β and CK1 have been shown to exert an inhibitory effect on Ci/GLI. Indeed, in absence of Hh, these three kinases phosphorylate Ci sequentially at three clusters of sites, where PKA functions as the priming kinase for subsequently phosphorylation by CK1 and GSK3β [40,41]. These phosphorylation events create the docking sites for the SCF^Slimb^ ubiquitin ligase, leading to Ci degradation [42,43]. Similar phosphorylation events mediated by these three kinases regulate the proteolytic cleavage of mammalian GLI2 and GLI3 into repressor forms by recruiting the β-TRCP E3 ubiquitin ligase [8,9,44,45]. Unlike GLI3, which is partially degraded with the production of GLI3 repressor form, GLI2 proteolysis leads to an almost complete degradation of the protein, suggesting that GLI3 is the major contributor of the repressor form.

PKA is also able to regulate GLI1 protein localization and activity through direct phosphorylation of GLI1 primarily at residue Thr374, which resides adjacent to the nuclear localization signal (NLS) [46]. A recent report showed that the pattern of GLI phosphorylation by PKA can negatively regulate GLI transcriptional activity in a graded manner; full phosphorylation by PKA at six conserved Serine residues drives GLI^R^ formation and blocks GLI^A^ formation, whereas full dephosphorylation of GLI at these clusters produces strong transcriptional activators [47]. Moreover, dual phosphorylation of SUFU by PKA and GSK3β at residues Ser342 and Ser346 stabilizes SUFU against HH signaling degradation [48]. In line with the negative regulation of the GLI by PKA, its inactivation in mice is able to induce an abnormal expansion of the skin stem cell compartment and to activate GLI1, leading to the rapid formation of BCC-like lesions [49].

CK1 can act as a positive regulator of Ci in presence of Hh. Indeed, CK1-mediated phosphorylation of Ci at multiple Ser/Thr-rich degrons protects Ci^A^ from degradation by the Cullin 3-based E3 ubiquitin ligase containing the BTB family protein HIB, allowing Ci^A^ to accumulate and to induce the expression of Hh target genes. Similarly, CK1 plays a conserved role in the regulation of vertebrate GLI^A^ activity by attenuating its degradation by SPOP, the vertebrate homolog of HIB [50]. However, another report suggests that CK1α associates to and negatively regulates GLI1 in mammalian cells. Indeed, CK1α overexpression enhances the proteasome-dependent degradation of GLI1, whereas CK1α pharmacological inhibition increases GLI1 protein levels. In addition, CK1α is able to phosphorylate GLI1, and this phosphorylation is further stimulated by the CK1α agonist pyrvinium [51]. It is unclear why CK1 plays opposite effects on GLI in mammalian, and further investigations are required to clarify this regulation.

GSK3β plays an inhibitory role on the GLI. GSK3β, GLI3 and SUFU can form a trimolecular complex, that stimulates GLI3 phosphorylation by GSK3β and, hence, GLI3 processing. Stimulation of C3H10T1/2 cells with Shh promotes the dissociation of the SUFU/GSK3β complex from GLI3, resulting in the blockade of GLI3 processing [52]. Consistent with the negative regulation of HH pathway by GSK3β, inactivation of GSK3 during mouse mammary gland development induces adenocarcinomas with activation of the HH pathway [53]. Moreover, GSK3β is involved in GLI2 ubiquitination and degradation, because genetic inhibition of GSK3β attenuates GLI2 ubiquitination, leading to increased GLI2 protein level [54]. Surprisingly, in pancreatic adenocarcinoma cells the GSK3β inhibitor lithium chloride has been shown to promote the ubiquitin-dependent proteasome degradation of GLI1. Furthermore, lithium inhibits pancreatic adenocarcinoma cell proliferation, blocking G1/S cell-cycle progression, and induces apoptosis. Lithium chloride synergizes with gemcitabine in reducing growth and tumorigenic potential of pancreatic adenocarcinoma cells [55]. These data are quite unexpected, because lithium chloride inhibits GSK3β and, therefore, it should upregulate GLI1 expression.

### 3.2. GRK2

The G protein-coupled receptor kinase 2 (GRK2) is a serine/threonine kinase that belongs to the GRK family. Most of the studies on the regulation of the HH pathway by GRK2 are in *Drosophila* and Zebrafish and very little is known about the role of GRK2 in mammalian. The *Drosophila* Gprk2 plays a role in the modulation of Hh signaling [56,57,58], by regulating Smo activation in both kinase-dependent and -independent manner. Gprk2 directly binds to Smo autoinhibitory domain (SAID) and phosphorylates Smo at Ser741/Thr742 to stabilize its active conformation and to promote Smo C-terminal dimerization. All these events are facilitated by PKA/CK1-mediated Smo phosphorylation, that recruits Gprk2. In turn, Hh enhances Gprk2 expression establishing a positive feedback loop [59]. Recent evidence showed that, when the Hh pathway is off, Gprk2 phosphorylates the E3 ubiquitin ligase Smurf at a serine cluster in its N-terminal, inducing the binding of Smurf to the SAID of Smo, which is ubiquitinated and degraded. On the other hand, Hh stimulation inhibits Smurf phosphorylation by Gprk2 and Smo degradation [60].

The mechanism of SMO regulation and activation in vertebrates seems to be similar to that described in *Drosophila* [61,62]. In mammalian cells and in Zebrafish embryos GRK2 positively regulates HH signaling [63,64,65]. Upon HH stimulation, GRK2 phosphorylates the C-terminus of SMO at six Ser/Thr clusters (S0–S5) together with CK1α, leading SMO to acquire an open active conformation and to move in the PC, where it induces HH pathway activation [62]. However, GRK2 is not necessary for SMO localization in the PC, but it is required for the response to HH signaling, because genetic and pharmacological inhibition of Grk2 reduces the expression of Hh target genes in Zebrafish embryos and mammalian cells [66,67]. A recent study suggested that GRK2 acts downstream of SMO but upstream of Gα_s_, likely at the level of Gα_s_-coupled GPCRs, such as GPR161, an attenuator of HH pathway [67]. Moreover, Grk2 plays also a role in controlling cell cycle during early development in Zebrafish by directly interacting with Ptch1, thus removing Ptch1-dependent inhibition on Cyclin B1, which can translocate into the nucleus and promote cell proliferation [68].

### 3.3. CK2

Casein kinase 2 (CK2) is a serine/threonine kinase that consists of subunits α and β. In *Drosophila,* CK2 has been shown to regulate both Smo and Ci, thus promoting Hh signaling. Indeed, CK2 phosphorylates Smo at multiple sites in its C-terminal cytoplasmic tail, inducing Smo activity. Nevertheless, the effect of CK2 on Smo are not as potent as those of PKA and CK1. CK2 acts also at the level of Ci, by preventing its ubiquitination and thus attenuating its proteosomal-dependent degradation [69].

The expression level of CK2α is tightly regulated in normal cells, whereas it is upregulated in a number of human cancers [70]. CK2 has been reported to positively regulate HH signaling in lung cancer cells. Indeed, CK2α inhibition decreases GLI1 expression and transcriptional activity, enhancing GLI1 degradation in A549 and H1299 lung cancer cell lines. In addition, genetic inhibition of CK2α leads to a reduction of the side population through downregulation of the ATP-binding transporter ABCG2, a putative target of HH signaling [71]. Consistently, silencing of CK2α inhibits migration and invasion and reduces the expression of GLI1 and PTCH1 in hepatocellular carcinoma Hep G2 cells [72]. Moreover, CK2α has been shown to positively modulate HH signaling in mesothelioma cells. Human mesothelioma samples show a positive correlation between GLI1 and CK2α expression, and CK2α genetic silencing or pharmacological inhibition with the small-molecule CK2α inhibitor CX-4945 reduces the expression and transcriptional activity of GLI1 [73].

CK2 was recently identified as the main driver of phosphorylation events during proliferation of cerebellar granule cell precursors (GCP), the cells of origin of medulloblastoma, in a phosphoproteome screening performed to discover new candidate drug targets in medulloblastoma. In the same manuscript authors showed that CK2 is required for HH signaling transduction and is critical for the stabilization and activity of GLI2 in medulloblastoma cells [74].

### 3.4. DYRK Family

Dual-specificity tyrosine phosphorylation-regulated kinases (DYRKs) are serine, threonine and tyrosine kinases containing a motif called DYRK-homology box. There are five members of mammalian DYRK, subdivided in two classes; DYRK1A and DYRK1B belong to class I, while class II consists of DYRK2, DYRK3 and DYRK4 [75].

Among the five DYRK members, DYRK1A, DYRK1B and DYRK2 are involved in the regulation of HH signaling. DYRK1A exerts an activating function on GLI1, promoting GLI1 nuclear translocation [76,77] through direct phosphorylation of a cluster of NLS located in the N-terminus (Ser102/104/130/132) [78] and at Ser408 [79]. However, a recent report showed that DYRK1A can induce GLI1 degradation through an indirect mechanism that engages the actin cytoskeleton and its regulators [78]. The dual role of DYRK1A in the regulation of HH signaling is likely due to interactions with different sets of partner proteins that elicit opposing effects.

Other reports suggest that also DYRK1B can play complex and dual roles in the modulation of HH pathway. DYRK1B can inhibit HH signaling, likely blocking GLI2 function and promoting GLI3^R^ formation by an unknown mechanism [80]. On the other hand, DYRK1B has been shown to enhance GLI1 activity and DYRK1B inhibition dampens GLI1 expression in both SMO-inhibitor sensitive and resistant cells [81]. Another study reported that DYRK1B can exert both positive and negative regulation on the HH pathway. It negatively interferes with SMO-elicited canonical HH signaling, while at the same time it promotes AKT-mediated GLI1 stability [82]. More recently, it has been shown that DYRK1B regulates HH-induced microtubule acetylation [83].

Finally, a kinome-wide screening identified DYRK2 among 480 kinases as a negative regulator of the HH pathway. The study showed that DYRK2 directly phosphorylates GLI2 at two conserved Serine residues (Ser385 and Ser1011) inducing its proteasome-dependent degradation [84]. In conclusion, the DYRK family plays a complex relationship with the HH pathway, with class I (DYRK1A and DYRK1B) having a dual role in the regulation of HH signaling, whereas DYRK2 has mainly an inhibitory function.

### 3.5. ERK1/2

The Mitogen-Activated Protein Kinase Extracellular signal-Regulated Kinase 1 and 2 (MAPK-ERK1/2) play pivotal role in controlling several cellular functions, including proliferation and cell cycle. ERK1 and ERK2 are highly similar serine/threonine kinases, which are activated by the upstream MEK1 and MEK2 in the RAS-RAF-MEK1/2-ERK1/2 signaling pathway [85]. Several studies reported a positive modulation of HH pathway by MEK1/2-ERK1/2 [86]. The first evidence came from a report showing that activated MEK1 enhances GLI1 expression and transcriptional activity. Supporting this positive regulation, co-expression of GLI1 and of a constitutively active mutant of MEK1 elicits a synergistic increase in GLI1 transcriptional activity, which is completely prevented by the MEK1/2 inhibitor PD98059 [87]. Authors identified a region in GLI1 N-terminal domain (amino acids 1–130) that senses the status of ERK1/2 signaling, as deletion of this region produces a transcriptionally active GLI1 protein with reduced response to MEK1 signaling. However, another kinase downstream of ERK1/2 is likely responsible for phosphorylation of the N-terminal region of GLI1, since direct phosphorylation of GLI1 by ERK1/2 was not demonstrated [87]. A putative MAPK consensus site within the N-terminus of GLI proteins was identified by another study [88]. Nevertheless, evidence of a direct phosphorylation is still lacking.

A number of reports have shown that the MAPK-ERK1/2 cascade can regulate HH signaling in several types of cancer. For instance, in melanoma cells oncogenic NRAS (NRAS^Q61K^) and HRAS (HRAS^V12G^) have been shown to activate GLI1 function, enhancing its transcriptional activity and nuclear localization. Mechanistically, both oncogenes counteract GLI1 cytoplasmic retention by SUFU. MEK1/2-ERK1/2 are the possible main effectors of RAS, because MEK1/2 inhibition reverses the effect of oncogenic RAS on GLI1 [27]. Consistently, in pancreatic cancer cells KRAS has been shown to increase GLI1 activity via MEK1/2-ERK1/2, and KRAS-mediated activation of GLI1 is suppressed with UO126, through decrease of GLI1 protein stability [89]. More recently, in multiple myeloma it has been shown that constitutively active MEK1 increases GLI2 stability and promotes its nuclear translocation, while reducing its ubiquitination. The kinase RSK2, which acts downstream of MEK1/2-ERK1/2 cascade, is able to mimic the effect of MEK1 on GLI2 stabilization. It is plausible to assume that MEK1/2-RSK2 stabilizes GLI2 by inhibiting GSK3β-mediated phosphorylation of GLI2, because MEK1 and RSK2 are not able to increase half-life of GLI2 lacking GSK3β phosphorylation sites [54].

### 3.6. AKT

AKT (or protein kinase B) is a serine/threonine kinase whose activation is regulated by the level of phosphatidylinositol-3-kinase (PI3K). The PI3K-AKT pathway plays a crucial role in many cellular processes, including cell cycle and apoptosis [90]. Several evidences suggest a positive regulation of the HH pathway by PI3K/AKT signaling, although a direct phosphorylation of the GLI by AKT has not yet been reported. A study showed that activation of PI3K/AKT potentiates SHH-induced GLI transcriptional activity, by antagonizing PKA-dependent GLI2 inactivation [91]. Activation of AKT signaling has been shown to enhance GLI1 nuclear localization and transcriptional activity in human melanoma cells, LNCaP prostate cancer cells and U87 glioma cells [27]. In addition, in renal cell carcinoma cells both GLI1 and GLI2 are activated by the PI3K/AKT signaling. Interestingly, a combination of GLI inhibitor GANT61 and AKT inhibitor perifosine synergistically suppresses renal cell carcinoma growth and induces apoptosis in vitro and in vivo [92]. Consistent with these findings, AKT1 activation appears to be required for BCC tumorigenesis in a SKH1-Ptch1^+/−^ mouse model that resembles features of patients with Basal Cell Nevus Syndrome. Interestingly, pharmacological inhibition of AKT decreases growth of BCC in this model [93]. Another report showed that *AKT1* is a direct target of GLI1 [94], suggesting the existence of a positive regulatory loop between AKT and HH signaling.

### 3.7. S6K1

p70 ribosomal protein S6 kinase 1 (S6K1) is a serine/threonine kinase that regulates many aspects of cellular biology, by controlling mRNA translation, ribosome biogenesis, cell growth and survival, authophagy, immune suppression and metabolism [95]. The mammalian target of rapamicin (mTOR)/S6K1 pathway has been proposed to mediates the development of esophageal adenocarcinoma (EAC) through GLI1 activation in a SMO-independent manner [96]. Authors found that in EAC cells the cytokine TNFα leads to activation of mTOR, which, in turn, phosphorylates its target S6K1. Thus, activated S6K1 directly phosphorylates GLI1 at Ser84 and enhances oncogenic GLI1 functions, by releasing GLI1 from its negative regulator SUFU [96]. In addition, mTOR/S6K1 signaling has been shown to contribute to radiotherapy-induced GLI1 activity in head and neck squamous cell carcinoma cell lines [97] and to enhance GLI1 expression in prostate cancer cell lines [98]. The mTOR/S6K1-GLI1 crosstalk appears to be context dependent, because in neuroblastoma cells S6K1 and GLI1 have been shown to exert proliferative effects through independent mechanisms and S6K1 does not affect GLI1 [99]. p70S6K2, another member of the S6K family, has also been shown to positively modulate the HH pathway in non-small cell lung cancer cells. Indeed, inhibition of p70S6K2 leads to a decrease of GLI1 protein level, likely through activation of GSK3β [100].

### 3.8. PKC Family

The protein kinase C (PKC) proteins are widely expressed serine/threonine kinases. They consist of three families: calcium-dependent conventional PKC (cPKC; isoforms α, βI, βII and γ); calcium-independent novel PKC (nPKC; isoforms δ, ε, η and θ); and calcium-independent atypical PKC (aPKC; isoforms ζ and ι/λ). All PKC have been shown to modulate HH signaling with different effects.

The role of both PKCα and PKCδ in the modulation of the HH pathway is controversial. Regarding PKCα, a report suggests that it negatively regulates GLI1, reducing its transcriptional activity without affecting its nuclear translocation [101]. However, another report shows that PKCα increases GLI1 transcriptional activity in NIH3T3 cells [102]. Similarly, constitutively active PKCδ increases GLI1 transcriptional activity, and its inhibition with Rottlerin decreases *PTCH1* mRNA level [101]. However, in human hepatoma Hep3B cells PKCδ has been shown to repress GLI1 transcriptional activity and nuclear localization, without affecting protein stability [102]. The mechanisms implicated in GLI1 regulation by PKCα and PKCδ are poorly understood; indeed, there are no evidence of a direct GLI1 phosphorylation by PKCα nor PKCδ.

aPKCι/λ has been identified as a GLI regulator in mouse BCC cells. aPKCι/λ directly phosphorylates GLI1 (likely at residues Ser243 and Thr304), resulting in enhanced DNA binding and transcriptional activation [103]. Furthermore, GLI1 promotes the transcription of *PRKCI*, the gene encoding for aPKCι, forming a positive GLI-aPKCι feedback loop in BCCs. Direct involvement of aPKC in regulation of HH signaling has been demonstrated also in *Drosophila*, where aPKC promotes phosphorylation and activation of Smo and Ci [104].

### 3.9. AMPK

5′-Adenosine monophosphate (AMP)-activated protein kinase (AMPK) is a serine/threonine kinase consisting of three subunits: α, β and γ. The AMPK activity is fine regulated by the AMP/ATP ratio and by other kinases. It is involved in mitochondrial activity and biogenesis, autophagy and cell proliferation, acting in a context-dependent manner [105]. Xu and colleagues described for the first time the negative role of AMPK in the regulation of HH pathway in hepatocellular carcinoma, where AMPK directly interacts with GLI1 and modulates its expression [106]. Activated AMPK phosphorylates GLI1 at Ser102, Ser408 and Thr1074, reducing its transcriptional activity and protein stability [107]. Moreover, AMPK increases GLI1 cytoplasmic localization and promotes its interaction with the E3 ubiquitin ligase β-TRCP, thus inducing GLI1 proteasomal degradation [108]. On the other hand, another report indicates that only phosphorylation at Ser408 on GLI1 by AMPK is crucial for GLI1 degradation and for the reduction of HH-driven cell growth in human medulloblastoma [109].

AMPK can play also a positive role in HH pathway. AMPK can act downstream of SMO to stimulate metabolic reprogramming towards glycolysis in adipocytes and increase glucose uptake [110]. Moreover, AMPK has been shown to mediate the effects of HH pathway on polyamine metabolism in cerebellar GCPs and in medulloblastoma. In response to HH activation, AMPK phosphorylates the zinc finger protein CNBP (Cellular Nucleic acid-Binding Protein) at Thr173, inducing its interaction with SUFU that prevents CNBP ubiquitination and degradation. In turn, CNBP enhances the translation of the enzyme ornithine decarboxylase (ODC), which induces the biosynthesis of polyamines, thereby controlling HH-dependent medulloblastoma growth in vivo and in vitro. The pharmacological inhibition of ODC with the irreversible inhibitor DFMO, inhibits HH-induced cell growth, supporting the use of DFMO as pharmacological agent for medulloblastoma treatment [111]. Recently, Zhang and colleagues provided another evidence of a pro-tumorigenic role of AMPK, demonstrating that the subunit AMPKα2 sustains SHH-driven mouse medulloblastoma tumorigenesis in vivo [112]. All together, these evidences suggest that AMPK can support or inhibit tumorigenesis depending on cellular context. Therefore, it is still debated the use of AMPK activators or inhibitors for cancer therapy.

### 3.10. ULK3

Unc-51 like kinase 3 (ULK3) is a serine/threonine protein kinase widely expressed, which takes part in several cellular processes, such as autophagy and cell division. ULK3 positively regulates HH signaling by direct phosphorylation of the GLI, enhancing their transcriptional activity and nuclear localization of GLI1 [113]. Interestingly, ULK3 can act also as a negative regulator of the HH pathway independently of its kinase activity. Indeed, in absence of SHH ligand, ULK3 physically interacts with SUFU through its kinase domain thus abolishing ULK3 ability to phosphorylate the GLI. In addition, the complex SUFU-ULK3 binds to GLI2 and promotes its proteolytic cleavage into the repressor form (GLI2^R^). On the other hand, in response to SHH, SUFU-ULK3 complex dissociates and ULK3 can act as positive regulator of GLI2 [114]. Recently, it has been found that the small molecule SU6668, an ATP competitive tyrosine and serine/threonine kinase inhibitor, inhibits ULK3 kinase activity; indeed, SU6668 reduces SHH-induced expression of the GLI in ULK3-dependent manner [115]. ULK3 is also involved in cancer-associated fibroblast (CAF) activation via the CSL protein, the transcriptional modulator of NOTCH pathway, which negatively regulates conversion of fibroblast into CAFs. Silencing of CSL increases the expression of its direct target ULK3, which in turn induces the activation of GLI1/2 and CAF effector genes. Therefore, ULK3 might represent a target to suppress CAFs activation and their tumor-enhancing properties [116].

### 3.11. Other Kinases

The HH signaling has been shown to be regulated also by other PKs. For instance, the serine/threonine kinase PFTK1 (or CDK14), a member of the CDC2-related protein kinase family, positively modulates the protein levels of SMO, PTCH1 and GLI1, thus controlling cell proliferation, invasion and EMT in colon cancer cells [117]. Another positive regulator of HH pathway is the integrin-linked kinase (ILK), a serine/threonine protein kinase implicated in regulation of various processes. ILK inhibition suppresses the localization of SMO in the PC and leads to a decrease *GLI1* and *GLI2* mRNA levels induced by SHH [118].

The right open reading frame kinase 3 (RIOK3) is a serine/threonine kinase involved in cell proliferation, migration and invasion in various cancers [119,120]. It has been identified through a kinase screen in human cells as a novel regulator of SUFU localization. RIOK3 induces SUFU nuclear accumulation and positively regulates HH signaling in a SUFU-dependent manner. Indeed, RIOK3 silencing decreases the expression of HH target genes, but this effect is lost in SUFU^−/−^ MEF cells. However, it is still unknown whether the regulation of SUFU by RIOK3 is direct or indirect [121]. The serine/threonine kinase NIMA-related kinase 2A (NEK2A) interacts with and phosphorylates SUFU at Thr225 and Ser352, increasing its protein stability through the inhibition of its proteasomal degradation, thus preventing GLI2 nuclear accumulation and transcriptional activity [122,123]. Moreover, NEK2A is also a direct target of GLI1 and GLI2, establishing a negative feedback loop between NEK2A and the GLI factors in mammalian cells [123].

The liver kinase B1 (LKB1, also known as STK11) is a serine/threonine kinase that has been found mutated and deregulated in several types of cancers, where it acts as a tumor suppressor [124,125]. LKB1 negatively modulates the expression of HH target genes both at mRNA and protein levels [126,127,128], acting downstream of SMO. LKB1 regulates also cerebellar development by controlling HH-mediated GCP proliferation [129]. The serine/threonine kinase polo-like kinase-1 (PLK1), which is involved in cell cycle regulation, phosphorylates mouse GLI1 at Ser481 (human GLI1 at Ser479) enhancing GLI1 nuclear exit and its interaction with SUFU, leading to the inhibition of HH signaling activity [130].

Protein Kinase G (PKG) is a serine/threonine-specific protein kinase that is activated by cyclic guanosine monophosphate (cGMP). In mammalian two PKG genes have been identified, encoding for PGK-I and PGK-II. Upon cGMP stimulation PKG-I has been shown to mediate the response to HH signaling in neural plate cells and in embryoid bodies [131,132]. The effect of cGMP/PKG on HH pathway is opposite to that of cAMP and PKA.

Recent evidence has shown an involvement of the mitogen-activated protein kinase kinase kinase (MAP3Ks) MEKK1, MEKK2 and MEKK3 in HH regulation. MEKK1 directly binds to and phosphorylates GLI1 on multiple residues at its C-terminal, leading to the inhibition of its transcriptional activity and enhancing its association with the 14-3-3 proteins. This association might explain the inhibitory effects on HH pathway by MEKK1 [133]. MEKK2 and MEKK3 act as sensors of growth factors, inflammatory and stress signaling and act upstream of the MEK5/ERK5 signaling [134]. MEKK2 and MEKK3 have been shown to phosphorylate GLI1 at multiple sites (Ser201, Ser204, Ser243, Ser968, Thr1074 and Ser1078), inhibiting its transcriptional activity and reducing its protein stability and nuclear localization. In addition, GLI1 phosphorylation by MEKK2/3 enhances the association between GLI1 and SUFU, which is required for cytoplasmic retention of GLI1 [135]. Moreover, MEKK1, MEKK2 and MEKK3 reduce HH-dependent medulloblastoma cells growth [133,135]. MAP3K10 acts positively on the HH pathway, by indirectly enhancing the activity of GLI2 through phosphorylation of DYRK2 and GSK3β [84]. In addition, MAP3K10 enhances pancreatic cancer cells growth probably by increasing GLI1 and GLI2 expression [136]. The c-Jun N-terminal kinases (JNK1-3) that belong to the MAPK family have been shown to phosphorylate GLI3 at Ser343 [88]. Another evidence of a connection between JNK and HH signaling comes from a report showing that pharmacological inhibition of JNK abolishes c-JUN phosphorylation and its interaction with GLI2, decreasing GLI-dependent keratinocyte cell cycle progression [137]. These findings suggest a positive modulation of HH pathway by JNK (Table 1).

## 4. Roles of PKs in Hedgehog Pathway Control: Implications for Cancer Therapy

In the last decade efforts to inhibit the HH pathway have been focused mainly on the development of SMO inhibitors. Several of these SMO antagonists, including vismodegib (GDC-0449), sonidegib (LDE-225), BMS-833923, saridegib (IPI-926), glasdegib (PF-04449913), taladegib (LY2940680), itraconazole and posaconazole, have shown efficacy in reducing growth of mouse xenografts and have been extensively investigated in a number of clinical trials in advanced cancers [138,139,140]. Among them, vismodegib and sonidegib, have been approved by the US Food and Drug Administration (FDA) and the European Medicines Agency (EMA) for treatment of locally advanced or metastatic BCC. However, despite promising preclinical results, the emergence of drug resistance and severe side effects have diminished the enthusiasm on SMO antagonists. As a consequence, new HH inhibitor development strategies have been focused on designing SMO inhibitors with novel chemical structures (e.g., MRT-92) [141,142] or discovering inhibitors that target the GLI transcription factors [143]. GANT58 and 61 are the first class of direct GLI antagonists to be identified, and both are able to interfere with GLI1- and GLI2-mediated transcription [144]. Glabrescione B is a newly identified natural compound that functions by binding GLI1 and impairing GLI1/DNA interaction [145]. ATO, an already FDA-approved drug for acute promyelocytic leukemia, has been shown to bind GLI1 protein, inhibiting its transcriptional activity, and to block ciliary accumulation of GLI2 [146,147]. Except for ATO, which is not a specific GLI inhibitor, none of the GLI antagonists has been tested in clinical trials.

**Figure 2:**
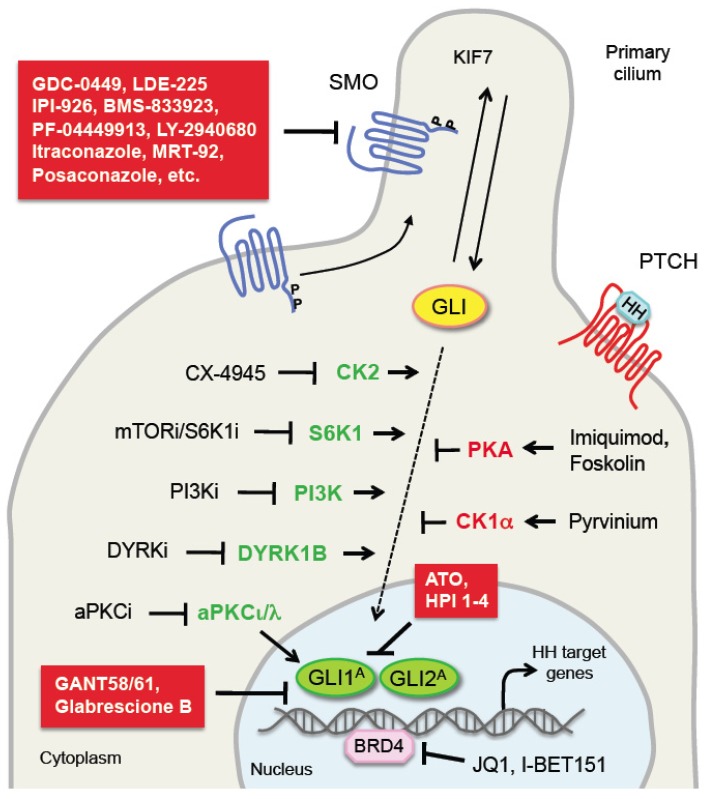
Targeting the Hedgehog pathway. Inhibition of the HH pathway by direct SMO and GLI inhibitors (red boxes) and by small molecules or drugs targeting protein kinases. Indicated are protein kinases that act as negative regulators (red) and positive regulators (green) of the GLI. For each protein kinase agonists and antagonists are reported. For details regarding the inhibitors and their target or mechanism of action see Table 1. Abbreviations: SMO, Smoothened; PTCH, Patched; KIF7, kinesin family member 7; HH, Hedgehog; GLI1/2^A^, GLI1/2 activators; CK1α, casein kinase 1α; CK2, casein kinase 2; PKA, protein kinase A; S6K1, ribosomal protein S6 kinase 1; PI3K, phosphatidylinositol-3-kinase; DYRK1B, dual specificity tyrosine-phosphorylation-regulated kinase 1B; aPKCι/λ, atypical protein kinase ι/λ; BRD4, bromodomain-containing protein 4.

In this review we have described the multifaceted roles that phosphorylation plays in HH pathway, focusing on cancer. Since the majority of PKs that phosphorylate and regulate components of the HH pathway can be modulated by antagonists/agonists, it is clear that their targeting may have important implications for anti-cancer therapy. Here, we will discuss examples of preclinical and clinical studies focusing on inhibition/activation of the above described HH-related PKs, alone or in combination with HH antagonists (Figure 2, Table 2).

Given the role of PKA in promoting Ci/GLI processing, compounds that act by targeting the HH signaling through activation of PKA are currently under clinical investigation. For instance, imiquimod, a synthetic nucleoside analogue of the imidazoquinoline family, was approved for topical treatment of small superficial BCCs. Imiquimod represses HH signaling by negatively modulating GLI activity in BCC and medulloblastoma cells. Mechanistically, imiquimod acts downstream of SMO enhancing PKA activity with consequent GLI2/3 phosphorylation and cleavage into GLI2/3 repressor forms [160]. Consistently, activation of PKA through the cAMP agonist forskolin is sufficient to inhibit HH pathway activity driven by oncogenic SMO (SMOA1 and W539L mutants) in vitro. Topical treatment of BCC with forskolin decreases tumor growth and reduces *GLI1* mRNA level in an inducible SMO-mutant BCC mouse model, providing evidence that forskolin inhibits growth of BCCs resistant to SMO inhibitors [161].

The anti-pinworm compound pyrvinium is an allosteric activator of CK1α. It acts by enhancing the degradation of the GLI in a CK1α-dependent manner. Pyrvinium is very potent and blocks HH signaling in the nanomolar concentration range, functioning also on vismodegib-resistant SMO and in settings of downstream pathway activation resulting from SUFU depletion. In vivo pyrvinium has been shown to attenuate growth of a Ptch^+/−^-derived medulloblastoma allografts and to reduce the expression of HH target genes [51].

aPKCι/λ is another potential therapeutic target, that was originally reported as an activator of GLI1 in BCC. Interestingly from a therapeutic point of view, vismodegib-resistant BCCs show high level of aPKCι/λ, and pharmacological inhibition of aPKC with PSI is able to suppress HH signaling and growth of resistant BCC cell lines [103]. A recent report showed that aPKCι/λ functions as a priming kinase for deacetylation of GLI1 by histone deacetylase 1 (HDAC1) [162], that is required for GLI transcriptional activation [167]. Combined targeting of HDAC1 and aPKC prevents GLI1 nuclear localization, and exerts cooperative effects in reducing growth of BCC cells in vitro, of patient-derived BCC explants ex vivo and of BCC in a mouse model. The same paper identified an ATP-competitive aPKC small-molecule inhibitor (CRT0329868) that shows a strong improvement in potency compared to previous generation inhibitors, with high bioavailability and efficacy in BCC. This study provided the first evidence that the aPKC-HDAC1 axis can be efficiently blocked in BCC using this aPKC antagonist in combination with the HDAC inhibitor vorinostat, providing an effective and novel therapeutic approach for BCC patients [162]. In addition, since this therapeutic approach inhibits nuclear GLI1, is also predicted to reduce the development of drug resistance.

A recent phosphoproteomic analysis identified the CK2 as a kinase critical for stabilization and activity of GLI2 and a promising therapeutic target in medulloblastoma. Pharmacological inhibition of CK2 with specific inhibitors decreases proliferation of primary SHH-type MB patient cells in vitro and inhibits growth of murine medulloblastoma that are resistant to SMO inhibitors. In particular, one of these CK2 antagonists (CX-4945) is able to inhibit both wild-type and mutant CK2, suggesting that this drug might prevent or delay acquired resistance [74]. This work has already directly led to clinical studies investigating the use of the CK2 inhibitor CX-4945 in patients with SHH-medulloblastoma.

S6K1 is another potential therapeutic target that directly phosphorylates GLI1 and promotes its oncogenic activity, suppressing the inhibitory effect of SUFU on GLI1. The report showed that EAC tissues express high levels of S6K1 and inhibition of its upstream activator mTOR with everolimus (RAD-001) enhances the effect of vismodegib in reducing EAC cell line proliferation and mouse xenograft growth [96]. These results support the use of combined therapy in cancers with active HH and mTOR/S6K1 pathways. Since specific S6K1 inhibitors, such as PF-4708671, are available, their effects on GLI activity should be tested [163].

Gene expression arrays of sonidegib-resistant mouse medulloblastoma have shown upregulation of PI3K, the upstream activator of S6K1. Combination therapy with sonidegib and the PI3K inhibitor NVP-BKM120 markedly delayed tumor resistance in allografted mouse [164]. In glioblastoma cells combination of PI3K inhibitor BKM120 and the SMO inhibitor LDE-225 signaling not only suppressed both pathways, but also inhibited S6K1 phosphorylation. Targeting both pathways induced mitotic catastrophe and tumor apoptosis, and decreased growth of PTEN-deficient glioblastomas in vitro and in orthotopic xenografts. Both drugs cross the blood-brain barrier and have acceptable toxicity profiles, providing a good therapeutic approach for glioblastoma treatment [165]. Similarly, the SMO antagonist sonidegib (LDE-225) has been shown to cooperate with the PI3K/mTOR inhibitor NVP-BEZ-235 to inhibit pancreatic cancer stem cell growth in vivo [166]. In addition, targeting both GLI and PI3K/mTOR signaling has shown a synergistic effect in reducing survival of primary cells from chronic lymphocytic leukemia patients, providing a further evidence of the efficacy of combined HH and PI3K signaling targeting [168].

Another study reported a novel small molecule DYRK inhibitor able to impair SMO-dependent and SMO-independent oncogenic GLI activity. Authors showed that genetic and pharmacological inhibition of DYRK1B reduces GLI1 expression in a number of cancer types, including human brain and pancreatic cancers, and mouse BCC cells. Interestingly, DYRK1B targeting represses GLI1 expression in both SMO-inhibitor sensitive and resistant cells [81].

## 5. Conclusions

Numerous kinases and phosphorylation events that regulate the HH signaling have been described in the last decade. Biochemical and functional analyses of kinases, such as PKA, CK1 and GSK3β, have provided important insights into HH signal transduction mechanisms, and begun to address how SMO and the GLI are regulated in physiological conditions. Despite these achievements, insights into the phosphorylation of several kinases, such as ERK1/2, AKT, PKCα and PKCδ, are still lacking. In addition, the full spectrum of phosphorylation of GLI1 and GLI2, the final effectors of the HH pathway, has only begun to be explored. Although PKA-mediated phosphorylation of the GLI has a predominant inhibitory effect on HH signaling, it is clear that the GLI undergo other important phosphorylation events that modulate their activities. For instance, S6K1 and aPKCι/λ directly phosphorylate and activate GLI1, respectively, in esophageal adenocarcinoma and BCC cells. It will be interesting to determine whether these phosphorylation events play a role in other types of cancer.

Given the dual role played by several kinases, such as PKA and CK1α, in the regulation of Smo/SMO and Ci/GLI, it is critical to understand how phosphorylation by these kinases is regulated. In addition, more work is needed to provide a clear picture of the exact role of these kinases in regulating the HH pathway in cancer, especially in those with non-canonical HH pathway activation. This aspect is particularly important in view of future therapeutic approaches targeting these kinases. Thus far, preclinical studies suggest that the most promising therapeutic targets may be S6K1, aPKC, CK2, PI3K and AKT. Targeting these kinases in combination with SMO or GLI antagonists may improve response rates and reduce resistance mechanisms, although more functional and preclinical studies are needed to define these relationships.

Finally, several kinases involved in phosphorylation events that modulate HH pathway have been identified, whereas most of the corresponding phosphatase are still unknown. Indeed, very few phosphatases have been described to regulate SMO, the GLI proteins or other HH pathway components; nevertheless, the molecular basis of their functions and, in particular, the specific residues are largely unknown. Therefore, in the near future efforts need to be directed towards the identification of phosphatases that play a role in the regulation of HH pathway.

## Figures and Tables

**Table 1:** Protein kinases involved in the regulation of HH pathway in mammalian.

Protein Kinases	Effect on HH Signaling	References
PKA	Inhibits GLI1, GLI2, GLI3	[8,9,44,45,46,47]
	Activates SMO	[39]
	Stabilizes SUFU	[48]
CK1	Activates/inhibits GLI	[50,51]
	Activates SMO	[62]
GSK3β	Inhibits GLI2, GLI3	[52,53,54]
	Stabilizes SUFU	[48]
GRK2	Activates SMO	[62]
CK2	Activates GLI1, GLI2	[71,72,73,74]
DYRK1A/1B	Activates/inhibits GLI	[76,77,78,79,80,81,82]
DYRK2	Inhibits GLI2	[84]
ERK1/2	Activates HH signaling	[27,87,88,89]
AKT	Activates HH signaling	[91,92,93]
S6K1	Activates GLI1	[96,97,98]
PKCα, δ	Activates/inhibits GLI1	[101,102]
aPKCι/λ	Activates GLI1	[103]
AMPK	Inhibits/Activates HH signaling	[106,107,108,109,110,111,112]
ULK3	Activates/inhibits GLI	[113,114,116]
PFTK1	Activates HH signaling	[117]
ILK	Activates HH signaling	[118]
RIOK3	Activates HH signaling	[121]
NEK2A	Increases SUFU stability repressing GLI2	[122,123]
LKB1	Inhibits HH signaling	[126,127,128]
PLK1	Inhibits GLI1	[130]
MEKK1	Inhibits GLI1	[133]
MEKK2/3	Enhances GLI1-SUFU association	[135]
MAP3K10	Activates GLI1 and GLI2	[84,136]
JNK	Activates GLI2 and GLI3	[88,137]

Abbreviations: HH, Hedgehog; SMO, Smoothened.

**Table 2:** Hedgehog pathway antagonists and other inhibitors mentioned in the text and in Figure 2.

Inhibitor	Pathway	Target/Mechanism	References
GDC-0449 (Vismodegib)	HH	SMO	[148]
LDE-225 (Sonidegib)	HH	SMO	[149]
IPI-926 (Saridegib)	HH	SMO	[150]
BMS-833923	HH	SMO	[151]
PF-04449913 (Glasdegib)	HH	SMO	[152]
LY2940680 (Taladegib)	HH	SMO	[153]
Itraconazole	HH	SMO	[154]
Posaconazole	HH	SMO	[155]
SANT1-4	HH	SMO	[156]
MRT-92	HH	SMO	[141,142]
GANT58-61	HH	Inhibit GLI1/2-mediated transcription	[144]
HPI1-4	HH	Modulate GLI activation	[157]
ATO	HH	Inhibits GLI1/2	[146,147]
Glabrescione B	HH	Interferes with GLI1/DNA binding	[145]
JQ1	BRD	Inhibits GLI1/2-mediated transcription	[158]
I-BET151	BRD	Inhibits GLI1/2-mediated transcription	[159]
Imiquimod	PKA	Inhibits GLI activity	[160]
Forskolin	PKA	PKA activation via cAMP	[161]
Pyrvinium	CK1α	Enhances CK1α-depend. GLI degradation	[51]
CX-4945	CK2	Inhibits CK2	[74]
PSI	aPKC	Antagonist	[103]
CRT0329868	aPKC	Antagonist	[162]
Everolimus (RAD-001)	mTOR/S6K1	Antagonist	[96]
PF-4708671	S6K1	Antagonist	[163]
NVP-BKM120	PI3K	Antagonist; inhibits also S6K1	[164,165]
NVP-BEZ235	PI3K/mTOR	Antagonist	[166]
DYRKi	DYRK1B	Antagonist	[81]

All SMO antagonists reported inhibit SMO by binding to its heptahelic transmembrane domain. Abbreviations: HH, Hedgehog; SMO, Smoothened; ATO, arsenic trioxide; BRD, bromodomain containing protein; PKA, protein kinase A; CK1α, casein kinase 1α; CK2, casein kinase 2; aPKC, atypical protein kinase C; mTOR/S6K1, mammalian target of rapamycin/S6 kinase 1; PI3K, phosphatidylinositol-3-kinase; DYRK1B, dual specificity tyrosine-phosphorylation-regulated kinase 1B.

## Abbreviations

AKT	Protein kinase B
AMPK	5′ Adenosine monophosphate (AMP)-activated protein kinase
aPKCι/λ	Atypical protein kinase Cι/λ
ATO	Arsenic trioxide
BCC	Basal cell carcinoma
BRD	Bromodomain
β-TRCP	β-transducin repeat containing protein
CAF	Cancer-associated fibroblast
Ci	Cubitus interruptus
CK1	Casein kinase 1
CK2	Casein kinase 2
DHH	Desert hedgehog
DYRK	Dual specificity tyrosine-phosphorylation-regulated kinase
ERK	Extracellular signal-regulated kinase
GCP	Granule cell precursors
GLI	Glioma-associated oncogene
GPCR	G-protein-coupled receptor
GRK2	G protein-coupled receptor kinase 2
GSK3β	Glycogen synthase kinase 3β
HH	Hedgehog
IHH	Indian hedgehog
ILK	Integrin-linked kinase
JNK	c-Jun N-terminal kinase
MAPK	Mitogen-activated protein kinase
mTOR	Mammalian target of rapamycin
NEK2A	NIMA-related kinase 2A
NLS	Nuclear localization signal
PC	Primary cilium
PI3K	Phosphatidylinositol-3-kinase
PK	Protein kinase
PKA	Protein kinase A
PKG	Protein kinase G
PLK1	Polo-like kinase 1
PTCH	Patched
RIOK3	Right open reading frame kinase 3
S6K1	S6 kinase 1
SAID	Smo autoinhibitory domain
SHH	Sonic hedgehog
SMO	Smoothened
SUFU	Suppressor of Fused
TNFα	Tumor necrosis factor α
ULK3	Unc-51 like kinase 3

## References

[1] Ingham P.W., McMahon A.P. (2001). Hedgehog signaling in animal development: Paradigms and principles. Genes Dev..

[2] Singh V., Ram M., Kumar R., Prasad R., Roy B.K., Singh K.K. (2017). Phosphorylation: Implications in Cancer. Protein J..

[3] Ardito F., Giuliani M., Perrone D., Troiano G., Lo Muzio L. (2017). The crucial role of protein phosphorylation in cell signaling and its use as targeted therapy. Int. J. Mol. Med..

[4] Chen Y., Jiang J. (2013). Decoding the phosphorylation code in Hedgehog signal transduction. Cell Res..

[5] Nüsslein-Volhard C., Wieschaus E. (1980). Mutations affecting segment number and polarity in Drosophila. Nature.

[6] Corbit K.C., Aanstad P., Singla V., Norman A.R., Stainier D.Y., Reiter J.F. (2005). Vertebrate Smoothened functions at the primary cilium. Nature.

[7] Rohatgi R., Milenkovic L., Scott M.P. (2007). Patched1 regulates hedgehog signaling at the primary cilium. Science.

[8] Pan Y., Bai C.B., Joyner A.L., Wang B. (2006). Sonic hedgehog signaling regulates Gli2 transcriptional activity by suppressing its processing and degradation. Mol. Cell. Biol..

[9] Wang B., Li Y. (2006). Evidence for the direct involvement of βTrCP in Gli3 protein processing. Proc. Natl. Acad. Sci. USA.

[10] Pan Y., Wang B. (2007). A novel protein-processing domain in Gli2 and Gli3 differentially blocks complete protein degradation by the proteasome. J. Biol. Chem..

[11] Kinzler K.W., Vogelstein B. (1990). The GLI gene encodes a nuclear protein which binds specific sequences in the human genome. Mol. Cell. Biol..

[12] Winklmayr M., Schmid C., Laner-Plamberger S., Kaser A., Aberger F., Eichberger T., Frischauf A.M. (2010). Non-consensus GLI binding sites in Hedgehog target gene regulation. BMC Mol. Biol..

[13] Hahn H., Wicking C., Zaphiropoulous P.G., Gailani M.R., Shanley S., Chidambaram A., Vorechovsky I., Holmberg E., Unden A.B., Gillies S. (1996). Mutations of the human homolog of Drosophila patched in the nevoid basal cell carcinoma syndrome. Cell.

[14] Taylor M.D., Liu L., Raffel C., Hui C.C., Mainprize T.G., Zhang X., Agatep R., Chiappa S., Gao L., Lowrance A. (2002). Mutations in SUFU predispose to medulloblastoma. Nat. Genet..

[15] Xie J., Murone M., Luoh S.M., Ryan A., Gu Q., Zhang C., Bonifas J.M., Lam C.W., Hynes M., Goddard A. (1998). Activating Smoothened mutations in sporadic basal-cell carcinoma. Nature.

[16] Kinzler K.W., Bigner S.H., Bigner D.D., Trent J.M., Law M.L., O’Brien S.J., Wong A.J., Vogelstein B. (1987). Identification of an amplified, highly expressed gene in a human glioma. Science.

[17] Northcott P.A., Nakahara Y., Wu X., Feuk L., Ellison D.W., Croul S., Mack S., Kongkham P.N., Peacock J., Dubuc A. (2009). Multiple recurrent genetic events converge on control of histone lysine methylation in medulloblastoma. Nat. Genet..

[18] Watkins D.N., Berman D.M., Burkholder S.G., Wang B., Beachy P.A., Baylin S.B. (2003). Hedgehog signalling within airway epithelial progenitors and in small-cell lung cancer. Nature.

[19] Thayer S.P., di Magliano M.P., Heiser P.W., Nielsen C.M., Roberts D.J., Lauwers G.Y., Qi Y.P., Gysin S., Fernández-del Castillo C., Yajnik V. (2003). Hedgehog is an early and late mediator of pancreatic cancer tumorigenesis. Nature.

[20] Feldmann G., Dhara S., Fendrich V., Bedja D., Beaty R., Mullendore M., Karikari C., Alvarez H., Iacobuzio-Donahue C., Jimeno A. (2007). Blockade of hedgehog signaling inhibits pancreatic cancer invasion and metastases: A new paradigm for combination therapy in solid cancers. Cancer Res..

[21] Berman D.M., Karhadkar S.S., Maitra A., Montes De Oca R., Gerstenblith M.R., Briggs K., Parker A.R., Shimada Y., Eshleman J.R., Watkins D.N. (2003). Widespread requirement for Hedgehog ligand stimulation in growth of digestive tract tumours. Nature.

[22] Karhadkar S.S., Bova G.S., Abdallah N., Dhara S., Gardner D., Maitra A., Isaacs J.T., Berman D.M., Beachy P.A. (2004). Hedgehog signalling in prostate regeneration, neoplasia and metastasis. Nature.

[23] Sanchez P., Hernández A.M., Stecca B., Kahler A.J., DeGueme A.M., Barrett A., Beyna M., Datta M.W., Datta S., Ruiz i Altaba A. (2004). Inhibition of prostate cancer proliferation by interference with SONIC HEDGEHOG-GLI1 signaling. Proc. Natl. Acad. Sci. USA.

[24] Varnat F., Duquet A., Malerba M., Zbinden M., Mas C., Gervaz P., Ruiz i Altaba A. (2009). Human colon cancer epithelial cells harbour active HEDGEHOG-GLI signalling that is essential for tumour growth, recurrence, metastasis and stem cell survival and expansion. EMBO Mol. Med..

[25] Clement V., Sanchez P., de Tribolet N., Radovanovic I., Ruiz i Altaba A. (2007). HEDGEHOG-GLI1 signaling regulates human glioma growth, cancer stem cell self-renewal, and tumorigenicity. Curr. Biol..

[26] Bar E.E., Chaudhry A., Lin A., Fan X., Schreck K., Matsui W., Piccirillo S., Vescovi A.L., DiMeco F., Olivi A. (2007). Cyclopamine-mediated hedgehog pathway inhibition depletes stem-like cancer cells in glioblastoma. Stem Cells.

[27] Stecca B., Mas C., Clement V., Zbinden M., Correa R., Piguet V., Beermann F., Ruiz i Altaba A. (2007). Melanomas require HEDGEHOG-GLI signaling regulated by interactions between GLI1 and the RAS-MEK/AKT pathways. Proc. Natl. Acad. Sci. USA.

[28] Yauch R.L., Gould S.E., Scales S.J., Tang T., Tian H., Ahn C.P., Marshall D., Fu L., Januario T., Kallop D. (2008). A paracrine requirement for hedgehog signalling in cancer. Nature.

[29] Becher O.J., Hambardzumyan D., Fomchenko E.I., Momota H., Mainwaring L., Bleau A.M., Katz A.M., Edgar M., Kenney A.M., Cordon-Cardo C. (2008). Gli activity correlates with tumor grade in platelet-derived growth factor-induced gliomas. Cancer Res..

[30] Dierks C., Grbic J., Zirlik K., Beigi R., Englund N.P., Guo G.R., Veelken H., Engelhardt M., Mertelsmann R., Kelleher J.F. (2007). Essential role of stromally induced hedgehog signaling in B-cell malignancies. Nat. Med..

[31] Hegde G.V., Munger C.M., Emanuel K., Joshi A.D., Greiner T.C., Weisenburger D.D., Vose J.M., Joshi S.S. (2008). Targeting of sonic hedgehog-GLI signaling: A potential strategy to improve therapy for mantle cell lymphoma. Mol. Cancer Ther..

[32] Pandolfi S., Stecca B. (2015). Cooperative integration between HEDGEHOG-GLI signalling and other oncogenic pathways: Implications for cancer therapy. Expert Rev. Mol. Med..

[33] Gu D., Xie J. (2015). Non-Canonical Hh Signaling in Cancer-Current Understanding and Future Directions. Cancers.

[34] Turnham R.E., Scott J.D. (2016). Protein kinase A catalytic subunit isoform PRKACA; History, function and physiology. Gene.

[35] Knippschild U., Gocht A., Wolff S., Huber N., Löhler J., Stöter M. (2005). The casein kinase 1 family: Participation in multiple cellular processes in eukaryotes. Cell. Signal..

[36] Doble B.W., Woodgett J.R. (2003). GSK-3: Tricks of the trade for a multi-tasking kinase. J. Cell Sci..

[37] Jia J., Tong C., Wang B., Luo L., Jiang J. (2004). Hedgehog signalling activity of Smoothened requires phosphorylation by protein kinase A and casein kinase I. Nature.

[38] Li S., Ma G., Wang B., Jiang J. (2014). Hedgehog induces formation of PKA-Smoothened complexes to promote Smoothened phosphorylation and pathway activation. Sci. Signal..

[39] Zhang B., Zhuang T., Lin Q., Yang B., Xu X., Xin G., Zhu S., Wang G., Yu B., Zhang T. (2019). Patched1-ArhGAP36-PKA-Inversin axis determines the ciliary translocation of Smoothened for Sonic Hedgehog pathway activation. Proc. Natl. Acad. Sci. USA.

[40] Jia J., Amanai K., Wang G., Tang J., Wang B., Jiang J. (2002). Shaggy/GSK3 antagonizes Hedgehog signalling by regulating Cubitus interruptus. Nature.

[41] Price M.A., Kalderon D. (2002). Proteolysis of the Hedgehog signaling effector Cubitus interruptus requires phosphorylation by Glycogen Synthase Kinase 3 and Casein Kinase 1. Cell.

[42] Jia J., Zhang L., Zhang Q., Tong C., Wang B., Hou F., Amanai K., Jiang J. (2005). Phosphorylation by double-time/CKIepsilon and CKIalpha targets cubitus interruptus for Slimb/beta-TRCP-mediated proteolytic processing. Dev. Cell.

[43] Smelkinson M.G., Kalderon D. (2006). Processing of the Drosophila hedgehog signaling effector Ci-155 to the repressor Ci-75 is mediated by direct binding to the SCF component Slimb. Curr. Biol..

[44] Wang B., Fallon J.F., Beachy P.A. (2000). Hedgehog-regulated processing of Gli3 produces an anterior/posterior repressor gradient in the developing vertebrate limb. Cell.

[45] Tempé D., Casas M., Karaz S., Blanchet-Tournier M.F., Concordet J.P. (2006). Multisite protein kinase A and glycogen synthase kinase 3beta phosphorylation leads to Gli3 ubiquitination by SCFbetaTrCP. Mol. Cell. Biol..

[46] Sheng T., Chi S., Zhang X., Xie J. (2006). Regulation of Gli1 localization by the cAMP/protein kinase A signaling axis through a site near the nuclear localization signal. J. Biol. Chem..

[47] Niewiadomski P., Kong J.H., Ahrends R., Ma Y., Humke E.W., Khan S., Teruel M.N., Novitch B.G., Rohatgi R. (2014). Gli protein activity is controlled by multisite phosphorylation in vertebrate Hedgehog signaling. Cell Rep..

[48] Chen Y., Yue S., Xie L., Pu X.H., Jin T., Cheng S.Y. (2011). Dual Phosphorylation of suppressor of fused (Sufu) by PKA and GSK3beta regulates its stability and localization in the primary cilium. J. Biol. Chem..

[49] Iglesias-Bartolome R., Torres D., Marone R., Feng X., Martin D., Simaan M., Chen M., Weinstein L.S., Taylor S.S., Molinolo A.A. (2015). Inactivation of a Gα(s)-PKA tumour suppressor pathway in skin stem cells initiates basal-cell carcinogenesis. Nat. Cell Biol..

[50] Shi Q., Li S., Li S., Jiang A., Chen Y., Jiang J. (2014). Hedgehog-induced phosphorylation by CK1 sustains the activity of Ci/Gli activator. Proc. Natl. Acad. Sci. USA.

[51] Li B., Fei D.L., Flaveny C.A., Dahmane N., Baubet V., Wang Z., Bai F., Pei X.H., Rodriguez-Blanco J., Hang B. (2014). Pyrvinium attenuates Hedgehog signaling downstream of smoothened. Cancer Res..

[52] Kise Y., Morinaka A., Teglund S., Miki H. (2009). Sufu recruits GSK3beta for efficient processing of Gli3. Biochem. Biophys. Res. Commun..

[53] Dembowy J., Adissu H.A., Liu J.C., Zacksenhaus E., Woodgett J.R. (2015). Effect of glycogen synthase kinase-3 inactivation on mouse mammary gland development and oncogenesis. Oncogene.

[54] Liu Z., Li T., Reinhold M.I., Naski M.C. (2014). MEK1-RSK2 contributes to Hedgehog signaling by stabilizing GLI2 transcription factor and inhibiting ubiquitination. Oncogene.

[55] Peng Z., Ji Z., Mei F., Lu M., Ou Y., Cheng X. (2013). Lithium inhibits tumorigenic potential of PDA cells through targeting hedgehog-GLI signaling pathway. PLoS ONE.

[56] Molnar C., Holguin H., Mayor F., Ruiz-Gomez A., de Celis J.F. (2007). The G protein-coupled receptor regulatory kinase GPRK2 participates in Hedgehog signaling in Drosophila. Proc. Natl. Acad. Sci. USA.

[57] Cheng S., Maier D., Neubueser D., Hipfner D.R. (2010). Regulation of smoothened by Drosophila G-protein-coupled receptor kinases. Dev. Biol..

[58] Maier D., Cheng S., Hipfner D.R. (2012). The complexities of G-protein-coupled receptor kinase function in Hedgehog signaling. Fly.

[59] Chen Y., Li S., Tong C., Zhao Y., Wang B., Liu Y., Jia J., Jiang J. (2010). G protein-coupled receptor kinase 2 promotes high-level Hedgehog signaling by regulating the active state of Smo through kinase-dependent and kinase-independent mechanisms in Drosophila. Genes Dev..

[60] Li S., Li S., Wang B., Jiang J. (2018). Hedgehog reciprocally controls trafficking of Smo and Ptc through the Smurf family of E3 ubiquitin ligases. Sci. Signal..

[61] Maier D., Cheng S., Faubert D., Hipfner D.R. (2014). A broadly conserved g-protein-coupled receptor kinase phosphorylation mechanism controls Drosophila smoothened activity. PLoS Genet..

[62] Chen Y., Sasai N., Ma G., Yue T., Jia J., Briscoe J., Jiang J. (2011). Sonic Hedgehog dependent phosphorylation by CK1α and GRK2 is required for ciliary accumulation and activation of smoothened. PLoS Biol..

[63] Chen W., Ren X.R., Nelson C.D., Barak L.S., Chen J.K., Beachy P.A., de Sauvage F., Lefkowitz R.J. (2004). Activity-dependent internalization of smoothened mediated by beta-arrestin 2 and GRK2. Science.

[64] Philipp M., Fralish G.B., Meloni A.R., Chen W., MacInnes A.W., Barak L.S., Caron M.G. (2008). Smoothened signaling in vertebrates is facilitated by a G protein-coupled receptor kinase. Mol. Biol. Cell.

[65] Meloni A.R., Fralish G.B., Kelly P., Salahpour A., Chen J.K., Wechsler-Reya R.J., Lefkowitz R.J., Caron M.G. (2006). Smoothened signal transduction is promoted by G protein-coupled receptor kinase 2. Mol. Cell. Biol..

[66] Zhao Z., Lee R.T., Pusapati G.V., Iyu A., Rohatgi R., Ingham P.W. (2016). An essential role for Grk2 in Hedgehog signalling downstream of Smoothened. EMBO Rep..

[67] Pusapati G.V., Kong J.H., Patel B.B., Gouti M., Sagner A., Sircar R., Luchetti G., Ingham P.W., Briscoe J., Rohatgi R. (2018). G protein-coupled receptors control the sensitivity of cells to the morphogen Sonic Hedgehog. Sci. Signal..

[68] Jiang X., Yang P., Ma L. (2009). Kinase activity-independent regulation of cyclin pathway by GRK2 is essential for zebrafish early development. Proc. Natl. Acad. Sci. USA.

[69] Jia H., Liu Y., Xia R., Tong C., Yue T., Jiang J., Jia J. (2010). Casein kinase 2 promotes Hedgehog signaling by regulating both smoothened and Cubitus interruptus. J. Biol. Chem..

[70] Trembley J.H., Wang G., Unger G., Slaton J., Ahmed K. (2009). Protein kinase CK2 in health and disease: CK2: A key player in cancer biology. Cell. Mol. Life Sci..

[71] Zhang S., Wang Y., Mao J.H., Hsieh D., Kim I.J., Hu L.M., Xu Z., Long H., Jablons D.M., You L. (2012). Inhibition of CK2α down-regulates Hedgehog/Gli signaling leading to a reduction of a stem-like side population in human lung cancer cells. PLoS ONE.

[72] Wu D., Sui C., Meng F., Tian X., Fu L., Li Y., Qi X., Cui H., Liu Y., Jiang Y. (2014). Stable knockdown of protein kinase CK2-alpha (CK2α) inhibits migration and invasion and induces inactivation of hedgehog signaling pathway in hepatocellular carcinoma Hep G2 cells. Acta Histochem..

[73] Zhang S., Yang Y.L., Wang Y., You B., Dai Y., Chan G., Hsieh D., Kim I.J., Fang L.T., Au A. (2014). CK2α, over-expressed in human malignant pleural mesothelioma, regulates the Hedgehog signaling pathway in mesothelioma cells. J. Exp. Clin. Cancer Res..

[74] Purzner T., Purzner J., Buckstaff T., Cozza G., Gholamin S., Rusert J.M., Hartl T.A., Sanders J., Conley N., Ge X. (2018). Developmental phosphoproteomics identifies the kinase CK2 as a driver of Hedgehog signaling and a therapeutic target in medulloblastoma. Sci. Signal..

[75] Becker W., Weber Y., Wetzel K., Eirmbter K., Tejedor F.J., Joost H.G. (1998). Sequence characteristics, subcellular localization, and substrate specificity of DYRK-related kinases, a novel family of dual specificity protein kinases. J. Biol. Chem..

[76] Mao J., Maye P., Kogerman P., Tejedor F.J., Toftgard R., Xie W., Wu G., Wu D. (2002). Regulation of Gli1 transcriptional activity in the nucleus by Dyrk1. J. Biol. Chem..

[77] Shimokawa T., Tostar U., Lauth M., Palaniswamy R., Kasper M., Toftgård R., Zaphiropoulos P.G. (2008). Novel human glioma-associated oncogene 1 (GLI1) splice variants reveal distinct mechanisms in the terminal transduction of the hedgehog signal. J. Biol. Chem..

[78] Schneider P., Bayo-Fina J.M., Singh R., Kumar Dhanyamraju P., Holz P., Baier A., Fendrich V., Ramaswamy A., Baumeister S., Martinez E.D. (2015). Identification of a novel actin-dependent signal transducing module allows for the targeted degradation of GLI1. Nat. Commun..

[79] Ehe B.K., Lamson D.R., Tarpley M., Onyenwoke R.U., Graves L.M., Williams K.P. (2017). Identification of a DYRK1A-mediated phosphorylation site within the nuclear localization sequence of the hedgehog transcription factor GLI1. Biochem. Biophys. Res. Commun..

[80] Lauth M., Bergström A., Shimokawa T., Tostar U., Jin Q., Fendrich V., Guerra C., Barbacid M., Toftgård R. (2010). DYRK1B-dependent autocrine-to-paracrine shift of Hedgehog signaling by mutant RAS. Nat. Struct. Mol. Biol..

[81] Gruber W., Hutzinger M., Elmer D.P., Parigger T., Sternberg C., Cegielkowski L., Zaja M., Leban J., Michel S., Hamm S. (2016). DYRK1B as therapeutic target in Hedgehog/GLI-dependent cancer cells with Smoothened inhibitor resistance. Oncotarget.

[82] Singh R., Dhanyamraju P.K., Lauth M. (2017). DYRK1B blocks canonical and promotes non-canonical Hedgehog signaling through activation of the mTOR/AKT pathway. Oncotarget.

[83] Singh R., Holz P.S., Roth K., Hupfer A., Meissner W., Müller R., Buchholz M., Gress T.M., Elsässer H.P., Jacob R. (2019). DYRK1B regulates Hedgehog-induced microtubule acetylation. Cell. Mol. Life Sci..

[84] Varjosalo M., Björklund M., Cheng F., Syvänen H., Kivioja T., Kilpinen S., Sun Z., Kallioniemi O., Stunnenberg H.G., He W.W. (2008). Application of active and kinase-deficient kinome collection for identification of kinases regulating hedgehog signaling. Cell.

[85] Roskoski R. (2012). ERK1/2 MAP kinases: Structure, function, and regulation. Pharmacol. Res..

[86] Rovida E., Stecca B. (2015). Mitogen-activated protein kinases and Hedgehog-GLI signaling in cancer: A crosstalk providing therapeutic opportunities?. Semin. Cancer Biol..

[87] Riobo N.A., Haines G.M., Emerson C.P. (2006). Protein kinase C-delta and mitogen-activated protein/extracellular signal-regulated kinase-1 control GLI activation in hedgehog signaling. Cancer Res..

[88] Whisenant T.C., Ho D.T., Benz R.W., Rogers J.S., Kaake R.M., Gordon E.A., Huang L., Baldi P., Bardwell L. (2010). Computational prediction and experimental verification of new MAP kinase docking sites and substrates including Gli transcription factors. PLoS Comput. Biol..

[89] Ji Z., Mei F.C., Xie J., Cheng X. (2007). Oncogenic KRAS activates hedgehog signaling pathway in pancreatic cancer cells. J. Biol. Chem..

[90] Brazil D.P., Yang Z.Z., Hemmings B.A. (2004). Advances in protein kinase B signalling: AKTion on multiple fronts. Trends Biochem. Sci..

[91] Riobó N.A., Lu K., Ai X., Haines G.M., Emerson C.P. (2006). Phosphoinositide 3-kinase and Akt are essential for Sonic Hedgehog signaling. Proc. Natl. Acad. Sci. USA.

[92] Zhou J., Zhu G., Huang J., Li L., Du Y., Gao Y., Wu D., Wang X., Hsieh J.T., He D. (2016). Non-canonical GLI1/2 activation by PI3K/AKT signaling in renal cell carcinoma: A novel potential therapeutic target. Cancer Lett..

[93] Kim A.L., Back J.H., Zhu Y., Tang X., Yardley N.P., Kim K.J., Athar M., Bickers D.R. (2016). AKT1 Activation is Obligatory for Spontaneous BCC Tumor Growth in a Murine Model that Mimics Some Features of Basal Cell Nevus Syndrome. Cancer Prev. Res..

[94] Agarwal N.K., Qu C., Kunkalla K., Liu Y., Vega F. (2013). Transcriptional regulation of serine/threonine protein kinase (AKT) genes by glioma-associated oncogene homolog 1. J. Biol. Chem..

[95] Guertin D.A., Sabatini D.M. (2007). Defining the role of mTOR in cancer. Cancer Cell.

[96] Wang Y., Ding Q., Yen C.J., Xia W., Izzo J.G., Lang J.Y., Li C.W., Hsu J.L., Miller S.A., Wang X. (2012). The crosstalk of mTOR/S6K1 and Hedgehog pathways. Cancer Cell.

[97] Gan G.N., Eagles J., Keysar S.B., Wang G., Glogowska M.J., Altunbas C., Anderson R.T., Le P.N., Morton J.J., Frederick B. (2014). Hedgehog signaling drives radioresistance and stroma-driven tumor repopulation in head and neck squamous cancers. Cancer Res..

[98] Yang H., Hu L., Liu Z., Qin Y., Li R., Zhang G., Zhao B., Bi C., Lei Y., Bai Y. (2017). Inhibition of Gli1-mediated prostate cancer cell proliferation by inhibiting the mTOR/S6K1 signaling pathway. Oncol. Lett..

[99] Diao Y., Rahman M.F., Villegas V.E., Wickström M., Johnsen J.I., Zaphiropoulos P.G. (2014). The impact of S6K1 kinase on neuroblastoma cell proliferation is independent of GLI1 signaling. BMC Cancer.

[100] Mizuarai S., Kawagishi A., Kotani H. (2009). Inhibition of p70S6K2 down-regulates Hedgehog/GLI pathway in non-small cell lung cancer cell lines. Mol. Cancer.

[101] Neill G.W., Ghali L.R., Green J.L., Ikram M.S., Philpott M.P., Quinn A.G. (2003). Loss of protein kinase C alpha expression may enhance the tumorigenic potential of Gli1 in basal cell carcinoma. Cancer Res..

[102] Cai Q., Li J., Gao T., Xie J., Evers B.M. (2009). Protein kinase Cdelta negatively regulates hedgehog signaling by inhibition of Gli1 activity. J. Biol. Chem..

[103] Atwood S.X., Li M., Lee A., Tang J.Y., Oro A.E. (2013). GLI activation by atypical protein kinase C ι/λ regulates the growth of basal cell carcinomas. Nature.

[104] Jiang K., Liu Y., Fan J., Epperly G., Gao T., Jiang J., Jia J. (2014). Hedgehog-regulated atypical PKC promotes phosphorylation and activation of Smoothened and Cubitus interruptus in Drosophila. Proc. Natl. Acad. Sci. USA.

[105] Mihaylova M.M., Shaw R.J. (2011). The AMPK signalling pathway coordinates cell growth, autophagy and metabolism. Nat. Cell Biol..

[106] Xu Q., Liu X., Zheng X., Yao Y., Wang M., Liu Q. (2014). The transcriptional activity of Gli1 is negatively regulated by AMPK through Hedgehog partial agonism in hepatocellular carcinoma. Int. J. Mol. Med..

[107] Li Y.H., Luo J., Mosley Y.Y., Hedrick V.E., Paul L.N., Chang J., Zhang G., Wang Y.K., Banko M.R., Brunet A. (2015). AMP-Activated Protein Kinase Directly Phosphorylates and Destabilizes Hedgehog Pathway Transcription Factor GLI1 in Medulloblastoma. Cell Rep..

[108] Zhang R., Huang S.Y., Li K.K., Li Y.H., Hsu W.H., Zhang G.J., Chang C.J., Yang J.Y. (2017). Dual degradation signals destruct GLI1: AMPK inhibits GLI1 through β-TrCP-mediated proteasome degradation. Oncotarget.

[109] Di Magno L., Basile A., Coni S., Manni S., Sdruscia G., D’Amico D., Antonucci L., Infante P., De Smaele E., Cucchi D. (2016). The energy sensor AMPK regulates Hedgehog signaling in human cells through a unique Gli1 metabolic checkpoint. Oncotarget.

[110] Teperino R., Amann S., Bayer M., McGee S.L., Loipetzberger A., Connor T., Jaeger C., Kammerer B., Winter L., Wiche G. (2012). Hedgehog partial agonism drives Warburg-like metabolism in muscle and brown fat. Cell.

[111] D’Amico D., Antonucci L., Di Magno L., Coni S., Sdruscia G., Macone A., Miele E., Infante P., Di Marcotullio L., De Smaele E. (2015). Non-canonical Hedgehog/AMPK-Mediated Control of Polyamine Metabolism Supports Neuronal and Medulloblastoma Cell Growth. Dev. Cell.

[112] Zhang H., Kuick R., Park S.S., Peabody C., Yoon J., Fernández E.C., Wang J., Thomas D., Viollet B., Inoki K. (2018). Loss of AMPKα2 Impairs Hedgehog-Driven Medulloblastoma Tumorigenesis. Int. J. Mol. Sci..

[113] Maloverjan A., Piirsoo M., Michelson P., Kogerman P., Osterlund T. (2010). Identification of a novel serine/threonine kinase ULK3 as a positive regulator of Hedgehog pathway. Exp. Cell Res..

[114] Maloverjan A., Piirsoo M., Kasak L., Peil L., Østerlund T., Kogerman P. (2010). Dual function of UNC-51-like kinase 3 (Ulk3) in the Sonic hedgehog signaling pathway. J. Biol. Chem..

[115] Piirsoo A., Kasak L., Kauts M.L., Loog M., Tints K., Uusen P., Neuman T., Piirsoo M. (2014). Protein kinase inhibitor SU6668 attenuates positive regulation of Gli proteins in cancer and multipotent progenitor cells. Biochim. Biophys. Acta.

[116] Goruppi S., Procopio M.G., Jo S., Clocchiatti A., Neel V., Dotto G.P. (2017). The ULK3 Kinase Is Critical for Convergent Control of Cancer-Associated Fibroblast Activation by CSL and GLI. Cell Rep..

[117] Zhu J., Liu C., Liu F., Wang Y., Zhu M. (2016). Knockdown of PFTAIRE Protein Kinase 1 (PFTK1) Inhibits Proliferation, Invasion, and EMT in Colon Cancer Cells. Oncol. Res..

[118] Barakat B., Yu L., Lo C., Vu D., De Luca E., Cain J.E., Martelotto L.G., Dedhar S., Sadler A.J., Wang D. (2013). Interaction of smoothened with integrin-linked kinase in primary cilia mediates Hedgehog signalling. EMBO Rep..

[119] Singleton D.C., Rouhi P., Zois C.E., Haider S., Li J.L., Kessler B.M., Cao Y., Harris A.L. (2015). Hypoxic regulation of RIOK3 is a major mechanism for cancer cell invasion and metastasis. Oncogene.

[120] Kimmelman A.C., Hezel A.F., Aguirre A.J., Zheng H., Paik J.H., Ying H., Chu G.C., Zhang J.X., Sahin E., Yeo G. (2008). Genomic alterations link Rho family of GTPases to the highly invasive phenotype of pancreas cancer. Proc. Natl. Acad. Sci. USA.

[121] Tariki M., Wieczorek S.A., Schneider P., Bänfer S., Veitinger S., Jacob R., Fendrich V., Lauth M. (2013). RIO kinase 3 acts as a SUFU-dependent positive regulator of Hedgehog signaling. Cell. Signal..

[122] Wang Y., Li Y., Hu G., Huang X., Rao H., Xiong X., Luo Z., Lu Q., Luo S. (2016). Nek2A phosphorylates and stabilizes SuFu: A new strategy of Gli2/Hedgehog signaling regulatory mechanism. Cell. Signal..

[123] Zhou F., Huang D., Li Y., Hu G., Rao H., Lu Q., Luo S., Wang Y. (2017). Nek2A/SuFu feedback loop regulates Gli-mediated Hedgehog signaling pathway. Int. J. Oncol..

[124] Wang Y.Q., Dai W.M., Chu X.Y., Yang B., Zhao M., Sun Y. (2014). Downregulation of LKB1 suppresses Stat3 activity to promote the proliferation of esophageal carcinoma cells. Mol. Med. Rep..

[125] Chen Y., Liu Y., Zhou Y., You H. (2019). Molecular mechanism of LKB1 in the invasion and metastasis of colorectal cancer. Oncol. Rep..

[126] Zhuang Z., Wang K., Cheng X., Qu X., Jiang B., Li Z., Luo J., Shao Z., Duan T. (2013). LKB1 inhibits breast cancer partially through repressing the Hedgehog signaling pathway. PLoS ONE.

[127] Xu P., Cai F., Liu X., Guo L. (2014). LKB1 suppresses proliferation and invasion of prostate cancer through hedgehog signaling pathway. Int. J. Clin. Exp. Pathol..

[128] Song K., Zheng G., Zhao Y. (2018). Liver kinase B1 suppresses growth of lung cancer cells through sonic hedgehog signaling pathway. Cell Biol. Int..

[129] Men Y., Zhang A., Li H., Jin Y., Sun X., Li H., Gao J. (2015). LKB1 Regulates Cerebellar Development by Controlling Sonic Hedgehog-mediated Granule Cell Precursor Proliferation and Granule Cell Migration. Sci. Rep..

[130] Zhang T., Xin G., Jia M., Zhuang T., Zhu S., Zhang B., Wang G., Jiang Q., Zhang C. (2019). The Plk1 kinase negatively regulates the Hedgehog signaling pathway by phosphorylating Gli1. J. Cell Sci..

[131] Robertson C.P., Gibbs S.M., Roelink H. (2001). cGMP enhances the sonic hedgehog response in neural plate cells. Dev. Biol..

[132] Christensen C., Zhang S., Roelink H. (2006). Inhibition of cGMP-dependent protein kinase reduces the response to sonic hedgehog in neuralized embryoid bodies. Stem Cells Dev..

[133] Antonucci L., Di Magno L., D’Amico D., Manni S., Serrao S.M., Di Pastena F., Bordone R., Yurtsever Z.N., Caimano M., Petroni M. (2019). Mitogen-activated kinase kinase kinase 1 inhibits hedgehog signaling and medulloblastoma growth through GLI1 phosphorylation. Int. J. Oncol..

[134] Cuevas B.D., Abell A.N., Johnson G.L. (2007). Role of mitogen-activated protein kinase kinase kinases in signal integration. Oncogene.

[135] Lu J., Liu L., Zheng M., Li X., Wu A., Wu Q., Liao C., Zou J., Song H. (2018). MEKK2 and MEKK3 suppress Hedgehog pathway-dependent medulloblastoma by inhibiting GLI1 function. Oncogene.

[136] An Y., Cai B., Chen J., Lv N., Yao J., Xue X., Tu M., Tang D., Wei J., Jiang K. (2013). MAP3K10 promotes the proliferation and decreases the sensitivity of pancreatic cancer cells to gemcitabine by upregulating Gli-1 and Gli-2. Cancer Lett..

[137] Laner-Plamberger S., Kaser A., Paulischta M., Hauser-Kronberger C., Eichberger T., Frischauf A.M. (2009). Cooperation between GLI and JUN enhances transcription of JUN and selected GLI target genes. Oncogene.

[138] Amakye D., Jagani Z., Dorsch M. (2013). Unraveling the therapeutic potential of the Hedgehog pathway in cancer. Nat. Med..

[139] Wu F., Zhang Y., Sun B., McMahon A.P., Wang Y. (2017). Hedgehog Signaling: From Basic Biology to Cancer Therapy. Cell Chem. Biol..

[140] Pietrobono S., Stecca B. (2018). Targeting the Oncoprotein Smoothened by Small Molecules: Focus on Novel Acylguanidine Derivatives as Potent Smoothened Inhibitors. Cells.

[141] Hoch L., Faure H., Roudaut H., Schoenfelder A., Mann A., Girard N., Bihannic L., Ayrault O., Petricci E., Taddei M. (2015). MRT-92 inhibits Hedgehog signaling by blocking overlapping binding sites in the transmembrane domain of the Smoothened receptor. FASEB J..

[142] Pietrobono S., Santini R., Gagliardi S., Dapporto F., Colecchia D., Chiariello M., Leone C., Valoti M., Manetti F., Petricci E. (2018). Targeted inhibition of Hedgehog-GLI signaling by novel acylguanidine derivatives inhibits melanoma cell growth by inducing replication stress and mitotic catastrophe. Cell Death Dis..

[143] Infante P., Alfonsi R., Botta B., Mori M., Di Marcotullio L. (2015). Targeting GLI factors to inhibit the Hedgehog pathway. Trends Pharmacol. Sci..

[144] Lauth M., Bergström A., Shimokawa T., Toftgård R. (2007). Inhibition of GLI-mediated transcription and tumor cell growth by small-molecule antagonists. Proc. Natl. Acad. Sci. USA.

[145] Infante P., Mori M., Alfonsi R., Ghirga F., Aiello F., Toscano S., Ingallina C., Siler M., Cucchi D., Po A. (2015). Gli1/DNA interaction is a druggable target for Hedgehog-dependent tumors. EMBO J..

[146] Kim J., Lee J.J., Kim J., Gardner D., Beachy P.A. (2010). Arsenic antagonizes the Hedgehog pathway by preventing ciliary accumulation and reducing stability of the Gli2 transcriptional effector. Proc. Natl. Acad. Sci. USA.

[147] Beauchamp E.M., Ringer L., Bulut G., Sajwan K.P., Hall M.D., Lee Y.C., Peaceman D., Ozdemirli M., Rodriguez O., Macdonald T.J. (2011). Arsenic trioxide inhibits human cancer cell growth and tumor development in mice by blocking Hedgehog/GLI pathway. J. Clin. Investig..

[148] Robarge K.D., Brunton S.A., Castanedo G.M., Cui Y., Dina M.S., Goldsmith R., Gould S.E., Guichert O., Gunzner J.L., Halladay J. (2009). GDC-0449-a potent inhibitor of the hedgehog pathway. Bioorg. Med. Chem. Lett..

[149] Pan S., Wu X., Jiang J., Gao W., Wan Y., Cheng D., Han D., Liu J., Englund N.P., Wang Y. (2010). Discovery of NVP-LDE225, a Potent and Selective Smoothened Antagonist. ACS Med. Chem. Lett..

[150] Tremblay M.R., Lescarbeau A., Grogan M.J., Tan E., Lin G., Austad B.C., Yu L.C., Behnke M.L., Nair S.J., Hagel M. (2009). Discovery of a potent and orally active hedgehog pathway antagonist (IPI-926). J. Med. Chem..

[151] Gendreau S.B., Hawkins D., Ho C.P., Lewin A., Lin T., Merchant A., Rowley R.B., Wang Q., Matsui W., Fargnoli J. (2009). Preclinical characterization of BMS-833923 (XL139), a hedgehog (HH) pathway inhibitor in early clinical development. In AACR-NCI-EORTC International Conference: Molecular Targets and Cancer Therapeutics. Mol. Cancer Ther..

[152] Munchhof M.J., Li Q., Shavnya A., Borzillo G.V., Boyden T.L., Jones C.S., LaGreca S.D., Martinez-Alsina L., Patel N., Pelletier K. (2011). Discovery of PF-04449913, a Potent and Orally Bioavailable Inhibitor of Smoothened. ACS Med. Chem. Lett..

[153] Bender M.H., Hipskind P.A., Capen A.R., Cockman M., Credille K.M., Gao H., Bastian J.A., Clay J.M., Lobb K.L., Sall D.J. (2011). Identification and characterization of a novel smoothened antagonist for the treatment of cancer with deregulated hedgehog signaling. Cancer Res..

[154] Kim J., Tang J.Y., Gong R., Kim J., Lee J.J., Clemons K.V., Chong C.R., Chang K.S., Fereshteh M., Gardner D. (2010). Itraconazole, a commonly used antifungal that inhibits Hedgehog pathway activity and cancer growth. Cancer Cell.

[155] Chen B., Trang V., Lee A., Williams N.S., Wilson A.N., Epstein E.H., Tang J.Y., Kim J. (2016). Posaconazole, a Second-Generation Triazole Antifungal Drug, Inhibits the Hedgehog Signaling Pathway and Progression of Basal Cell Carcinoma. Mol. Cancer Ther..

[156] Chen J.K., Taipale J., Young K.E., Maiti T., Beachy P.A. (2002). Small molecule modulation of Smoothened activity. Proc. Natl. Acad. Sci. USA.

[157] Hyman J.M., Firestone A.J., Heine V.M., Zhao Y., Ocasio C.A., Han K., Sun M., Rack P.G., Sinha S., Wu J.J. (2009). Small-molecule inhibitors reveal multiple strategies for Hedgehog pathway blockade. Proc. Natl. Acad. Sci. USA.

[158] Tang Y., Gholamin S., Schubert S., Willardson M.I., Lee A., Bandopadhayay P., Bergthold G., Masoud S., Nguyen B., Vue N. (2014). Epigenetic targeting of Hedgehog pathway transcriptional output through BET bromodomain inhibition. Nat. Med..

[159] Long J., Li B., Rodriguez-Blanco J., Pastori C., Volmar C.H., Wahlestedt C., Capobianco A., Bai F., Pei X.H., Ayad N.G. (2014). The BET bromodomain inhibitor I-BET151 acts downstream of smoothened protein to abrogate the growth of hedgehog protein-driven cancers. J. Biol. Chem..

[160] Wolff F., Loipetzberger A., Gruber W., Esterbauer H., Aberger F., Frischauf A.M. (2013). Imiquimod directly inhibits Hedgehog signalling by stimulating adenosine receptor/protein kinase A-mediated GLI phosphorylation. Oncogene.

[161] Makinodan E., Marneros A.G. (2012). Protein kinase A activation inhibits oncogenic Sonic hedgehog signalling and suppresses basal cell carcinoma of the skin. Exp. Dermatol..

[162] Mirza A.N., Fry M.A., Urman N.M., Atwood S.X., Roffey J., Ott G.R., Chen B., Lee A., Brown A.S., Aasi S.Z. (2017). Combined inhibition of atypical PKC and histone deacetylase 1 is cooperative in basal cell carcinoma treatment. JCI Insight.

[163] Pearce L.R., Alton G.R., Richter D.T., Kath J.C., Lingardo L., Chapman J., Hwang C., Alessi D.R. (2010). Characterization of PF-4708671; a novel and highly specific inhibitor of p70 ribosomal S6 kinase (S6K1). Biochem. J..

[164] Buonamici S., Williams J., Morrissey M., Wang A., Guo R., Vattay A., Hsiao K., Yuan J., Green J., Ospina B. (2010). Interfering with resistance to smoothened antagonists by inhibition of the PI3K pathway in medulloblastoma. Sci. Transl. Med..

[165] Gruber Filbin M., Dabral S.K., Pazyra-Murphy M.F., Ramkissoon S., Kung A.L., Pak E., Chung J., Theisen M.A., Sun Y., Franchetti Y. (2013). Coordinate activation of Shh and PI3K signaling in PTEN-deficient glioblastoma: New therapeutic opportunities. Nat. Med..

[166] Sharma N., Nanta R., Sharma J., Gunewardena S., Singh K.P., Shankar S., Srivastava R.K. (2015). PI3K/AKT/mTOR and sonic hedgehog pathways cooperate together to inhibit human pancreatic cancer stem cell characteristics and tumor growth. Oncotarget.

[167] Canettieri G., Di Marcotullio L., Greco A., Coni S., Antonucci L., Infante P., Pietrosanti L., De Smaele E., Ferretti E., Miele E. (2010). Histone deacetylase and Cullin3-REN(KCTD11) ubiquitin ligase interplay regulates Hedgehog signalling through Gli acetylation. Nat. Cell Biol..

[168] Kern D., Regl G., Hofbauer S.W., Altenhofer P., Achatz G., Dlugosz A., Schnidar H., Greil R., Hartmann T.N., Aberger F. (2015). Hedgehog/GLI and PI3K signaling in the initiation and maintenance of chronic lymphocytic leukemia. Oncogene.

